# Impairment of nuclear F-actin formation and its relevance to cellular phenotypes in Hutchinson-Gilford progeria syndrome

**DOI:** 10.1080/19491034.2020.1815395

**Published:** 2020-09-20

**Authors:** Yuto Takahashi, Shogo Hiratsuka, Nanako Machida, Daisuke Takahashi, Junpei Matsushita, Pavel Hozak, Tom Misteli, Kei Miyamoto, Masahiko Harata

**Affiliations:** a Laboratory of Molecular Biology, Graduate School of Agricultural Science, Tohoku University, Sendai, Japan; b Institute of Molecular Genetics of the Czech Academy of Sciences, Prague, Czech Republic; c National Cancer Institute, National Institutes of Health, Bethesda, MD, USA; d Laboratory of Molecular Developmental Biology, Faculty of Biology-Oriented Science and Technology, Kindai University, Wakayama, Japan

**Keywords:** Nuclear actin, lamin, nuclear organization, Hutchinson-Gilford progeria syndrome, progerin, gene expression

## Abstract

Hutchinson-Gilford progeria syndrome (HGPS) is a premature aging disorder caused by a mutation of lamin A, which contributes to nuclear architecture and the spatial organization of chromatin in the nucleus. The expression of a lamin A mutant, named progerin, leads to functional and structural disruption of nuclear organization. Since progerin lacks a part of the actin-binding site of lamin A, we hypothesized that nuclear actin dynamics and function are altered in HGPS cells. Nuclear F-actin is required for the organization of nuclear shape, transcriptional regulation, DNA damage repair, and activation of Wnt/β-catenin signaling. Here we show that the expression of progerin decreases nuclear F-actin and impairs F-actin-regulated transcription. When nuclear F-actin levels are increased by overexpression of nuclear-targeted actin or by using jasplakinolide, a compound that stabilizes F-actin, the irregularity of nuclear shape and defects in gene expression can be reversed. These observations provide evidence for a novel relationship between nuclear actin and the etiology of HGPS.

## Introduction

Hutchinson-Gilford progeria syndrome (HGPS) is a rare childhood disease occurring in one in four to eight million newborns [1]. In HGPS patients, scleroderma, aging of skin and bone defects are generally observed [2]. With the progression of the disease, some patients suffer other HGPS-associated symptoms such as accelerated arteriosclerosis, kidney failure, and cardiovascular problems [2–4]. Most patients die from heart attacks and strokes because of atherosclerotic disease and myocardial infarction [2]. Aging progresses faster in HGPS patients than normal and the mean age of death of HGPS patients is around 13 years. There is no cure or effective treatment to date.

HGPS is caused by a *de novo* C1824T mutation in the human *LMNA* gene encoding the nuclear architectural protein lamin A [5]. Lamin A belongs to the type V intermediate filament family, and its polymer is present at the inner nuclear membrane. Lamin A filaments form a meshwork and contribute to the organization of nuclear structure and the maintenance of chromatin organization and genome stability [6]. The occurrence of the single base substitution in *LMNA* leads to the mis-splicing of exon 11 in mRNA. As a result, a truncated version of lamin A, named progerin, is produced in HGPS patient cells [7].

Progerin lacks 50 amino acid residues in its carboxy- (C-) terminal region [7], and its expression causes extensive changes in nuclear lamina structure and functions which result in abnormal nuclear architecture and epigenetic dysregulation. For example, distorted nuclear shape and a reduction of heterochromatin are observed in HGPS cells [8–10] and epigenetic marks such as chemical modifications of histone and DNA are altered in HGPS cells [8,11]. In addition, genomic instability and increased DNA damage is observed [9,12]. Gene expression is also affected in HGPS cells. Notably, Wnt/β-catenin signaling, known to be a fundamental pathway, is diminished in HGPS cells [13,14]. It has been suggested that the suppression of Wnt/β-catenin signal activity and decreased levels of Wnt/β-catenin-responsive gene expression is the cause of bone defects in HGPS [15,16]. At present, it is thought that multiple nuclear dysfunctions caused by the expression of progerin facilitate the progression of HGPS symptoms and accelerated cellular senescence [5].

Actin is present both in the cytoplasm and the nucleus [17]. As in the cytoplasm, nuclear actin exists in globular (G-) and filamentous (F-) forms, and multiple nuclear proteins regulate actin dynamics [18–20]. Recent studies have shown that nuclear actin has various roles in genome function and nuclear organization [19,21]. Nuclear F-actin is required for the organization of nuclear shape after cell division. Interestingly, the formation of nuclear F-actin is observed at the exit of cells from mitosis and is involved in nuclear expansion and proper chromatin organization in the nucleus [22]. Nuclear F-actin also regulates gene expression, for example, by enhancing the expression of Wnt/β-catenin target genes through binding to β-catenin [23]. It is also required for the relocalization of DNA double-strand break (DSB) sites in the nucleus and the progression of the DNA damage repair processes [24,25]. Nuclear G-actin also has roles in gene expression. Multiple chromatin remodeling and histone modification complexes contain G-actin [26]. Nuclear G-actin also regulates the transcriptional activity of serum response factor (SRF) via interaction with megakaryoblastic leukemia 1 (MKL1), which is a co-activator of the SRF transcription factor [27,28].

Nuclear actin directly binds to lamin [29] with the interaction first described in 1998 [30]. Subsequent research using a yeast two-hybrid assay identified two actin-binding sites in lamin A [31]. The first is formed by amino acids 461–531 while the second is formed by amino acids 564–646. Because of the HGPS mutation, progerin lacks the 50 amino acid residues between 606 and 656, and consequently lacks a part of actin-binding site 2 (606–646). Indeed, an in vitro assay showed that the deletion results in a diminished level of interaction between nuclear actin and progerin relative to wild-type lamin A [29]. Consistent with the reported interaction between actin and lamin A, lamin A has been shown to be involved in the regulation of actin dynamics [32,33]. It is notable that knockdown of *LMNA* diminishes nuclear F-actin [34], suggesting a role for lamin A in F-actin formation in the nucleus. A recent study has shown that nuclear F-actin formation induced by GPCR signaling initiates at the inner nuclear membrane (INM) [35]. Importantly, because progerin lacks one of the two actin-binding sites in lamin A [29], it is possible that the dynamics and functions of nuclear actin are impaired in HGPS cells.

The cellular phenotype of HGPS can be induced by artificial expression of progerin in cultured cells, and this system has been utilized for analyzing molecular mechanisms in the etiology of HGPS [36–39]. This system has the advantage of being able to temporally control the induction of HGPS phenotypes and the ability to observe the same cell lines with or without progerin induction. For the induction of progerin expression, a tet-on system has been used. This system utilizes a reverse tetracycline transactivator (rtTA) and a tetracycline response element (TRE), whereby rtTA bound to TRE induces progerin expression in the presence of tetracycline [40,41].

In this study, using an inducible model of HGPS of progerin expression and probes for nuclear actin, we show that nuclear F-actin is decreased in progerin-expressing cells. Consistently, gene expression induced by nuclear F-actin is repressed by the expression of progerin. Importantly, some cellular HGPS phenotypes can be rescued by an increase of nuclear F-actin levels. These results demonstrate a tight association between HGPS etiology and nuclear F-actin.

## Materials and methods

### Cell culture and plasmids

Human dermal fibroblasts (HDFs) that express GFP-progerin upon treatment with doxycycline (Dox; 1 μg/ml) was used as model system for HGPS cells [37,38]. HDFs that express Dox inducible GFP-lamin A were used as control cells. Both these cells are immortalized by stable expression of human telomerase reverse transcriptase (hTERT). These cells were cultured in Gibco MEM (Gibco, 10370–021) supplemented with 2 mM L-glutamine, 1 mM sodium pyruvate (Gibco), non-essential amino acids (Gibco, 11140–050), 15% fetal bovine serum, 100 U/ml penicillin, and 100 μg/ml streptomycin at 37 °C in 5% CO_2_/air humidified atmosphere. The number of cultured cells was determined using the trypan blue exclusion method and a Thoma hemocytometer in accordance with the manufacturer’s protocol.

NIH3T3 cells stably expressing nAC-mCherry were established using the Jump-In system, a method to establish cell strains which stably express exogenous genes in mammalian cells. This system uses a PhiC31 pseudo-attP site in the mammalian genome. The integration of the exogenous gene is performed through the recombination of pseudo-attP sites and a PhiC attB site which is mediated by PhiC31 integrase. For this system, a pJTI-nAC-mCherry plasmid and pJTI-PhiC31 plasmid were transfected into NIH3T3 cells. pJTI-nAC-mCherry has a PhiC attB site upstream of the nAC-mCherry coding sequence. pJTI-PhiC31 plasmid was transfected into cells to express PhiC31 integrase. Transfection was performed using FuGENE HD Transfection Reagent (Promega) in accordance with the manufacturer’s protocol. 24 hours after transfection, selection of NIH3T3 cells stably expressing nAC-mCherry by addition of puromycin (5 µg/ml) was performed. After selection, living NIH3T3 cells were picked, and fluorescence from nAC-mCherry was confirmed.

Descriptions of mCherry-actin (expressing mCherry fused β-actin), mCherry-NLS-actin (expressing NLS attached β-actin tagged by mCherry), mCherry-NLS-G13R actin (expressing mCherry-NLS-G13R actin mutant), EYFP-actin (expressing EYFP fused β-actin), and EYFP-NLS-actin (expressing NLS attached β-actin tagged by EYFP) are in previous reports [23,42]. The nAC-mCherry plasmid construct expresses an NLS-attached actin-chromobody tagged by mCherry [34]. These constructs were transfected into 1 × 10^6^ cells by electroporation using a NEPA21 Type Ⅱ Super Electroporator (NEPA GENE) 72 hours after the addition of Dox (except for the experiments using FTI). The amount of construct was 10 μg each for sole-transfection or 5 μg for each for co-transfection.

### Imaging analysis

For quantitative analysis of the subcellular fluorescence signal intensity, Trainable WEKA segmentation, a plugin tool for Fiji, was used. Using this tool, binary masks of whole cellular regions were generated based on mCherry fluorescence signal. The binary mask of the nuclear region was generated based on the fluorescence signal of 4’, 6-diamidino-2-phenylindole (DAPI) using the same tool. After the whole cellular region and nuclear region were determined by each binary mask, we measured mCherry fluorescence signal intensity of each region by Fiji. After the measurement, the ratio of nuclear/cytoplasmic fluorescence signal intensity was calculated as the relative nuclear fluorescence signal intensity.

### Quantification of nuclear morphology

To quantitatively assess nuclear morphological irregularity, NII_Plugin, a plugin tool for Fiji, was used. According to the manufacturer’s protocol [43], we calculated nuclear roundness, radius ratio, and Area/Box. Nuclear roundness was determined by the formula described in the protocol [43]. The radius ratio is the ratio between the maximum and the minimum radius of the nucleus. Area/Box is the ratio between the area of the nucleus and the area of its bounding box. Using these values, an index called NII value was calculated. We optimized NII value for our research. The following is the formula used to calculate NII value: radius ratio + nuclear roundness – Area/Box.

### Immunofluorescence staining and microscopy

Cells grown on glass slides at 50–80% confluency were used for immunofluorescence staining. Cells were fixed with 4% paraformaldehyde in phosphate-buffered saline (PBS) for 15 min at room temperature. The cells were permeabilized with 0.5% Triton X-100 in PBS for 10 min at room temperature. Nuclei in permeabilized cells were stained with DAPI (Invitrogen) for 5 min at room temperature. To observe the subcellular localization of G-actin, permeabilized cells were treated with DNaseI (Invitrogen, D12371) in PBS overnight at 4 °C before staining with DAPI. DNaseI is conjugated with Alexa Fluor 594. For observing nuclear γH2AX foci, permeabilized cells were treated with an anti-γH2AX monoclonal primary antibody (Millipore, DAM1493341) overnight at 4 °C. After treatment with the primary antibody, cells were stained by an anti-mouse IgG secondary antibody tagged by Alexa Fluor 350 (Thermo Fisher Scientific, A-11045). To distinguish mitotic nuclei from interphase nuclei, we detected α-tubulin with an anti-α-tubulin monoclonal antibody (Sigma-Aldrich, T9026) overnight at 4 °C after permeabilization. Subsequently to the primary antibody reaction, cells were stained by an anti-mouse IgG secondary antibody tagged by Alexa Fluor 594 (Thermo Fisher Scientific, A-11005). The slides were mounted with Vectashield Mounting Medium (Vector Laboratories, H-1000). The cells were observed using IX83 wide-field fluorescence microscopy (Olympus) with a 60x lens (Olympus, UPlanSApo, numerical aperture of 1.35). Images were captured with an ORCA Flash 4.0 LT PLUS Digital CMOS camera (Hamamatsu, Model C11440-42U30).

### Time-lapse imaging and quantification using fluorescence microscopy

NIH3T3 cells stably expressing nAC-mCherry were seeded on each glass-bottom dish (IWAKI, No. 3911–035). The concentration of NIH3T3 cells seeded on a glass-bottom dish was 1.0 × 10^5^ cells/dish. After 24 hours of seeding, Hoechst 33342 (Hoechst, Dojindo) was applied to the culture medium (500 ng/ml) to stain nuclei for an hour. After staining with Hoechst, jasplakinolide (Adipogen Life Sciences) diluted in dimethyl sulfoxide (DMSO) was added, and time-lapse imaging microscopy started immediately (IX83 wide-field fluorescence microscopy, Olympus). Time-lapse images were taken every 15 minutes. Three fields were taken during each experiment. The observation was continued for 15–18 hours. To capture images, a 60x lens (Olympus, UPlanSApo, numerical aperture of 1.35) and ORCA Flash 4.0 LT PLUS Digital CMOS camera (Hamamatsu, Model C11440-42U30) were used.

### Quantitative real-time PCR analysis

Total RNA was extracted from cells using the Qiagen RNeasy Mini kit according to the manufacturer’s instructions. RNA was reverse-transcribed to cDNA using the PrimeScript RT reagent Kit (TaKaRa). Quantitative real-time PCR (qRT-PCR) was carried out using the SYBR-Green PCR kit (Applied Biosystems) in a total volume of 10 µl. The transcript of glyceraldehyde-3-phosphate dehydrogenase gene (*GAPDH*) was used for the normalization of the values by the ΔΔCt method. The sequences of the oligonucleotide primers used for this analysis are shown below:

*GAPDH*; Fw, 5’-TGCACCACCAACTGCTTAGC-3’; Rv, 5’-GGCATGGACTGTGGTCATGAC-3’

*TCF-1*; Fw, 5’-TGACCTCTCTGGCTTCTACT-3’; Rv, 5’-TTGATGGTTGGCTTCTTGGC-3’

*ACTB*; Fw, 5’-GCCGTCTTCCCCCTCCATCG-3’; Rv, 5’-TCTTGCTCTGGGCCTCGTC-3’

*Cfl1*; Fw, 5’-TCTCGTCTTCTGCGGCTCTC-3’; Rv, 5’-TCCAGGATGATGTTCTTCTTGTC-3’

*mDia2*; Fw, 5’-GCGGGAAAAGGACTTCAGTAT-3’; Rv, 5’-TCTGTCGGCTTCTCTTAAGACTTC-3’

*FMN2*; Fw, 5’-GCGAACGCTGTTGGAGAAG-3’; Rv, 5’-CTGATTACACGGTTCCCTGAAG-3’

Wiskott-Aldrich Syndrome protein (WASP); Fw, 5’-AAGACCTTGTGGCTACCCCT-3’;

WASP; Rv, 5’-AGCACACAGCCCCACAATGCTC-3’

*LMNB1*; Fw, 5’-AAGCTGTCTCCAAGCCCTTCTTC-3’; Rv, 5’-AACCCTCTTCCGCTTTCCTCTAG-3’

*LMNB2*; Fw, 5’-GGTGATGCGTGAGAATGAGAATG-3’; Rv, 5’-CCCCTGTTGGTGGAAAAGATC-3’

Emerin; Fw, 5’-AGAGGAGTGCAAGGATAGGGAACG-3’;

Emerin; Rv, 5’-GACATAAAAGAGGTGGAGGAGGAAG-3’

*LMNA*; Fw, 5’-ACCCCGCTGAGTACAACCTGC-3’; Rv, 5’-GCAGAAGAGCCAGAGGAGATGG-3’

*GFP*; Fw, 5’-GCAGAAGAACGGCATCAAGGTG-3’; Rv, 5’-GGGTGCTCAGGTAGTGGTTGTC-3’

### Western blotting analysis

Whole-cell extracts were prepared in cell lysis buffer (10 mM Tris-HCl [pH 8.0], 150 mM NaCl, 0.5 mM EDTA, 0.5% NP-40, protease inhibiter [Roche]) with sonication (30 s ON/90 s OFF × 6 times) using Bioruptor (Cosmobio). Protein samples were separated by SDS-PAGE and transferred onto a PVDF membrane using a semi-dry transfer system (Trans-Blot Turbo, BioRad). The transferred membrane was blocked with TBS-T (10 mM Tris-HCl [pH 8.0], 150 mM NaCl, 0.05% Tween20) containing 5% BSA. The membrane was incubated 1 hour in a primary antibody diluted with Can Get Signal solution 1 (TOYOBO), and washed with TBS-T three times. The membrane was then incubated for one hour in a secondary antibody diluted with Can Get Signal solution 2 (TOYOBO). Washing three times with TBS-T, signals were developed with ECL prime western blotting detection reagents (GE Healthcare), and subsequently detected using the Chemidoc analyzer (BioRad). Primary antibodies were used at the following dilution rate. Anti-actin antibody (Thermo Fisher Scientific, MS-1295-P1): 1/1000. Anti-H3 antibody (Abcam, ab1791): 1/10,000. Anti-GFP antibody (MBL, 598): 1/5000. Anti-rabbit IgG (Promega, W4011) or anti-mouse IgG (Promega, W4021) were used as secondary antibodies at 1/10,000 dilution.

### Dual-luciferase reporter assay

Dual-luciferase assays were performed using the Dual-Luciferase Reporter Assay System (Promega) according to the manufacturer’s instructions. The TCF/LEF reporter plasmid construct (Promega) was transfected into 1 × 10^6^ cells by electroporation, and the measurement performed after 24 hours. The pRL-TK renilla luciferase construct (Promega) was used as an internal control for transfection efficiency. Both firefly and renilla luciferase activity were measured using a GloMax® 20/20 Luminometer (Promega). The ratio of firefly to renilla luciferase activities was used as the TCF/LEF promoter activity.

### Senescence associated-β-galactosidase (SA-β-gal) detection

SA-β-gal in progerin-expressing cells was detected using the Cellular Senescence Plate Assay Kit – SPiDER-βGal (Dojindo, SG05). Detection was performed according to the user’s protocol. The fluorescence signal was measured using FP-8200 Spectrofluorometer (JASCO) at 30 °C. The wavelength of excitation light was 530.0 nm, and SA-β-gal was detected at 548.0–549.0 nm. The value of the fluorescence signal was normalized by the number of living cells counted before the SA-β-gal measurement.

### FTI and jasplakinolide treatment

As a farnesyltransferase inhibitor (FTI), lonafarnib (Sigma-Aldrich) dissolved in DMSO was added to the culture medium together with Dox. Transfection of plasmids into the cells was performed 24 hours after the addition of FTI and Dox. The FTI/Dox treatment was continued for 24 hours after the transfection, and then the cells were analyzed. For vehicle control, DMSO was solely added to the culture medium. Jasplakinolide was diluted in DMSO. For analyses using progerin-expressing cells, jasplakinolide and Dox were simultaneously added to the cell culture medium after 72 hours of Dox induction.

### Statistical analyses

For statistical analyses, at least three independent experiments were conducted, and values expressed as means. All statistical analyses were performed using Microsoft Excel. The p-value (*P*) was calculated using an unpaired or pair-sample two-tailed Student’s t-test. A p-value of less than 0.05 was defined as a statistically significant difference between two data items. The numbers of independent experiments and analyzed samples are indicated in the figure legends.

## Results and discussion

### Nuclear F-actin formation is decreased in progerin-expressing cells

**Figure 1.**
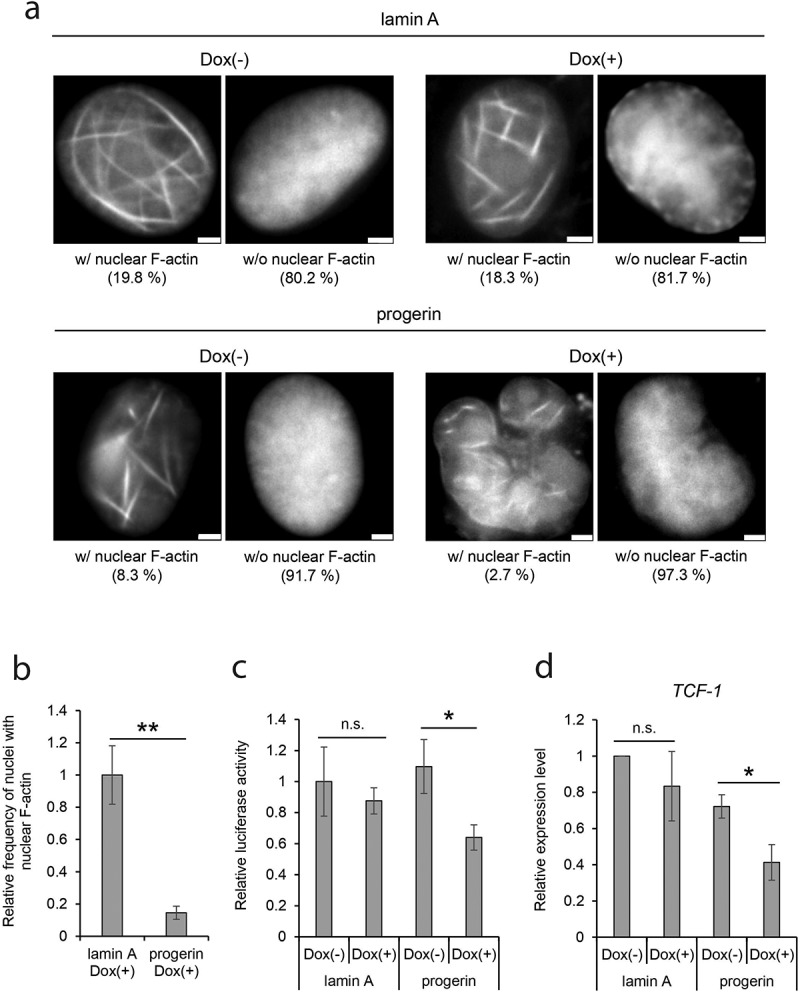
The reduction of nuclear F-actin in progerin-expressing cells. (a) Human dermal fibroblast (HDF) cells containing GFP-lamin A or GFP-progerin cDNA regulated by a Dox-inducible promoter were transfected with nAC-mCherry, and the cells cultured with or without Dox for 96 hr. Typical mCherry images corresponding to the nuclei with F-actin (w/F-actin) and to nuclei without F-actin (w/o F-actin) are shown. The numbers under panels indicate the percentage of cells possessing nuclear F-actin in each cell group. Scale bar, 2 μm. (b) Relative frequency of nuclei with nuclear F-actin. The mean value of lamin A-Dox(+) was defined as 1.0. Data shown are mean ± standard error measurement (SEM) of six independent experiments. For the quantification, 735 cells (lamin A-Dox(+)) and 771 cells (progerin-Dox(+)) were analyzed. (c) Relative transcriptional activity of the TCF/LEF promoter measured by luciferase-assay. The mean measured value in lamin A-Dox(-) was assigned as 1.0. Data shown are mean ± SEM of four independent experiments. (d) The relative expression level of *TCF-1* in control and progerin-expressing cells. The expression level was measured by qRT-PCR and was normalized with respect to that of *GAPDH* gene. The expression level of *TCF-1* in lamin A-Dox(-) was assigned as 1.0. Data shown are mean ± SEM of four independent experiments. n.s., not significant; *, *P* < 0.05; **, *P* < 0.01.

To analyze the behavior of nuclear actin in HGPS cells, we employed human dermal fibroblast (HDF) cells expressing GFP-lamin A or GFP-progerin cDNA under a Dox-inducible promoter (Figure 1(a) and Suppl. Fig. S1A) [37,38]. Typically, irregularities in nuclear shape, impairment of DNA damage repair, and decline of cell growth are observed in HGPS patient cells [9,44]. As previously demonstrated, expression of GFP-progerin upon Dox-treatment induced many of these defects [9,44], including irregular nuclear shape (Figure 1(a) and Suppl. Fig. S1A, progerin-Dox(+)). We quantitatively assessed the irregularity of nuclear shape using the Nuclear Irregularity Index (NII, see Materials and Methods). The NII values revealed that nuclear irregularity is significantly increased by the induction of GFP-progerin, but not by GFP-lamin A as a control (Suppl. Fig. S1B). To assess DNA damage, we observed γH2AX foci in cells with or without GFP-progerin induction. The number of γH2AX foci was increased by induction of progerin, indicating the accumulation of DNA damage by GFP-progerin (Suppl. Fig. S1C). Cell growth also declined upon induction of GFP-progerin (Suppl. Fig. S1D). Based on these observations and those from previous reports [9,44], we utilized the cells expressing GFP-progerin upon Dox-induction as a model system for HGPS cells (progerin-Dox(+)) in this study. Uninduced progerin-integrated cells (progerin-Dox(-)) were used as a control. HDF cells expressing GFP-lamin A upon Dox-treatment (lamin A-Dox(+)) and cells not expressing GFP-lamin A (lamin A-Dox(-)) were used as further controls.

To analyze nuclear F-actin by fluorescent microscopy, we transfected progerin-Dox(+) cells and control cells with a construct for expression of nAC-mCherry, which consists of an actin-chromobody tagged with a nuclear localization signal (NLS) and mCherry [34]. The efficiency of the transfection of these cells was consistent between the groups of cells (Suppl. Fig. S2A). The appearance of fibrous fluorescent structures in the nucleus enables us to identify cells containing nuclear F-actin (Figure 1(a)). Induction of progerin in the cells for 96 hours led to a decrease of the number of cells with nuclear F-actin (Figure 1(a), bottom panels, 8.3% in progerin-Dox(-) vs. 2.7% in progerin-Dox(+)). These results suggest that progerin decreases the formation of nuclear F-actin. In other control cells integrated with GFP-lamin A cDNA (Figure 1(a), top panels), the number of cells with nuclear F-actin was higher than in progerin-integrated cells (Figure 1(a), left panels, 19.8% in lamin A-Dox(-) vs. 8.3% in progerin-Dox(-)). A small amount of GFP-progerin was continuously expressed even in the absence of Dox (Suppl. Fig. S3), and it is likely that this leaky expression of progerin leads to the reduction of nuclear F-actin in all cell lines. Consistently, the number of cells exhibiting nuclear F-actin was constant even after the expression of GFP-lamin A (Figure 1(a), top panels, 19.8% in lamin A-Dox(-) vs. 18.3% in lamin A-Dox(+)). Since the formation of nuclear F-actin is reported to be transiently induced at the exit from mitosis [22], we checked the possibility that the number of mitotic cells in progerin-expressing cells affects nuclear F-actin. When mitotic cells were counted, the relative number of mitotic cells was not significantly changed between tested cell groups (Suppl. Fig. S2B). Together with the statistically significant reduction in the number of nuclei possessing nuclear F-actin in progerin-expressing cells (Figure 1(b)), these observations suggest the possibility that nuclear F-actin is decreased in HGPS cells.

We previously demonstrated that nuclear F-actin facilitates the binding of Wnt/β-catenin to chromatin and activates Wnt/β-catenin-targeted promoters [23]. To probe for functional consequences of the decrease of nuclear F-actin in progerin-expressing cells, we examined the activity of the TCF/LEF promoter, which is activated by Wnt/β-catenin-binding. A luciferase assay revealed that the transcriptional activity of the TCF/LEF promoter is reduced upon the expression of progerin (Figure 1(c), progerin-Dox(-) vs. progerin-Dox(+)). In contrast, the expression of lamin A does not affect TCF/LEF promoter activity (Figure 1(c), lamin A-Dox(-) vs. lamin A-Dox(+)). We then analyzed the expression of endogenous *TCF-1*, a Wnt/β-catenin-inducible gene [45]. Quantitative RT-PCR revealed that the expression level of *TCF-1* was significantly reduced by the expression of progerin but not lamin A (Figure 1(d)). Interestingly, the expression of Wnt/β-catenin-inducible genes is reported to be decreased in cells of HGPS patients [13,14]. We suggest that the decrease of nuclear F-actin by progerin expression contributes to the misregulation of genes in HGPS cells.

### The effect of progerin on nuclear actin properties other than polymerization

**Figure 2.**
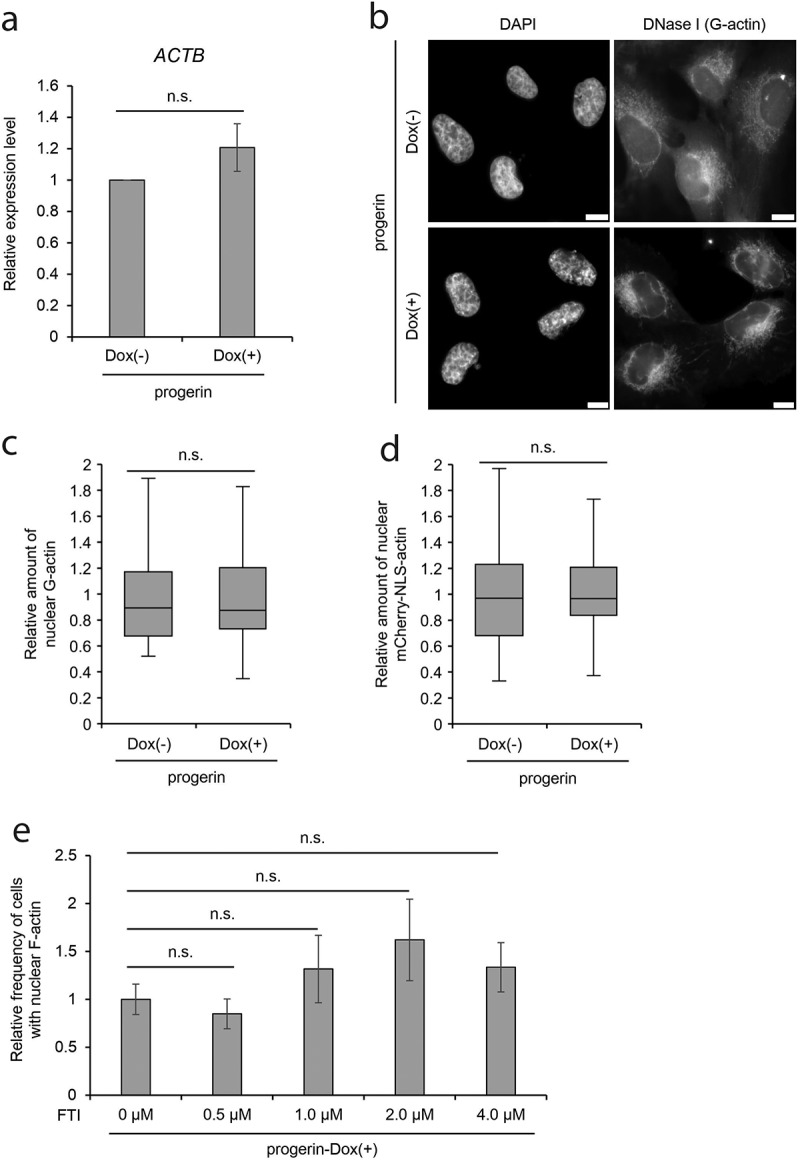
Indices of nuclear actin in progerin-expressing cells. (a) The relative expression level of *ACTB* gene in progerin-expressing cells. The expression level was measured by qRT-PCR and was normalized with respect to that of *GAPDH* gene. The expression level of *ACTB* in progerin-Dox(-) cells was assigned as 1.0. Data shown are mean ± SEM of three independent experiments. (b) Images of progerin-expressing cells stained by DAPI and DNase I conjugated with Alexa Fluor 594 (DNase I). Scale bar, 10 μm. (c) The relative amount of nuclear G-actin was defined as the ratio of nuclear to the cytoplasmic signal intensity of DNase I-staining of progerin-expressing cells. Data are shown as a box plot. The mean value of progerin-Dox(-) cells was assigned as 1.0. Error bars indicate standard deviation (SD) of three independent experiments. For quantification, 88 cells (progerin-Dox(-)) and 87 cells (progerin-Dox(+)) were analyzed. More than 20 cells were analyzed in each independent experiment. (d) The relative amount of nuclear mCherry-NLS-actin was defined as the ratio of nuclear to the cytoplasmic signal intensity of mCherry-fluorescence in progerin-expressing cells and shown as in C. Error bars indicate SD of three independent experiments. For quantification, 63 cells and 83 cells were analyzed. More than 20 cells were analyzed in each independent experiment. (e) Relative frequency of nuclei possessing nuclear F-actin in progerin-expressing cells treated with FTI. The mean value of progerin-expressing cells without FTI treatment was defined as 1.0. Data shown are mean ± SEM of three (4.0 μM FTI) and four (0, 0.5, 1.0, and 2.0 μM FTI) independent experiments. For quantification, 459 cells (0 μM FTI), 429 cells (0.5 μM FTI), 533 cells (1.0 μM FTI), 506 cells (2.0 μM FTI), and 335 cells (4.0 μM FTI) were analyzed. n.s., not significant.

To investigate mechanisms of the decrease in nuclear F-actin in progerin-expressing cells, we next analyzed aspects of nuclear actin properties other than its polymerization into F-actin. We find that the actin gene (*ACTB*) was expressed at a similar level in progerin-uninduced and -induced cells (Figure 2(a)), as were genes for the actin-binding proteins (ABPs) cofilin1, mDia2, formin2, and WASP (Suppl. Fig. S4A). The level of actin protein was further evaluated by western blotting and found to be unchanged after the Dox induction of progerin (Suppl. Fig. S4B). Subsequently we analyzed the amount of nuclear G-actin in progerin-expressing cells using Alexa Fluor 594 conjugated-Deoxyribonuclease I (DNase I), an established G-actin probe (Figure 2(b)) [27,32,46,47]. The intensity of the fluorescence for G-actin in the nucleus, relative to that in the cytoplasm, was not significantly changed in progerin-expressing cells (Figure 2(c), progerin-Dox (+)). Combined with our observation that the cellular level of actin protein is unaffected by the expression of progerin, we conclude that the amount of nuclear G-actin is unaffected by the induction of HGPS phenotypes.

To test the possibility that progerin affects the nuclear import of proteins, including actin-binding proteins (ABPs) [18], and hence indirectly affects the formation of F-actin, we analyzed the nuclear import activity of a nuclear localization signal (NLS) derived from SV40. Consistent with a previous report [48], the nuclear accumulation of NLS-tagged actin was not changed in progerin-expressing cells (Figure 2(d), progerin-Dox(+)), suggesting that nuclear import is not disturbed by the expression of progerin. These observations suggest that progerin affects the polymerization of nuclear F-actin, without affecting actin import.

### The actin-biding region of lamin A is required for the maintenance of nuclear F-actin

In addition to the lack of its C-terminal residues, which include the actin-binding region, progerin is characterized by farnesylation of its C-terminus [7]. In the case of lamin A, newly translated pre-lamin A (the precursor of lamin A) is farnesylated at its C-terminus. Subsequently, the farnesylated C-terminal region is deleted by the endoprotease Zmpste24/FACE-1 [7]. However, Zmpste24/FACE-1 does not recognize progerin since its target site is contained in the deleted region in progerin, and therefore, the farnesylated C-terminus is retained in progerin [7]. To investigate the involvement of the farnesylation of progerin in the decrease of nuclear F-actin, we treated progerin-expressing cells with a farnesyl transferase inhibitor (FTI). The FTI treatment did not rescue the reduction of nuclear F-actin in progerin-expressing cells (Figure 2(e)), suggesting that the deletion of the actin-binding region in progerin, but not its farnesylation, is predominantly involved in the decrease of nuclear F-actin.

Together with a previous study, which demonstrated that the knockdown of *LMNA* diminishes nuclear F-actin formation [34], our observations suggest that the actin-binding region of lamin A contributes to the formation of nuclear F-actin and that progerin, lacking a part of the actin-binding region, interferes with nuclear F-actin formation. Previously, an *in vitro* rhodamine-phalloidin bundling assay has consistently shown that progerin facilitates actin polymerization more weakly than lamin A [29]. Given our results, we propose that the dynamics of nuclear actin are maintained by lamin A, and that the expression of progerin disturbs its homeostasis in HGPS cells.

### Nuclear F-actin rescues cellular phenotypes of progerin-expressing cells

**Figure 3.**
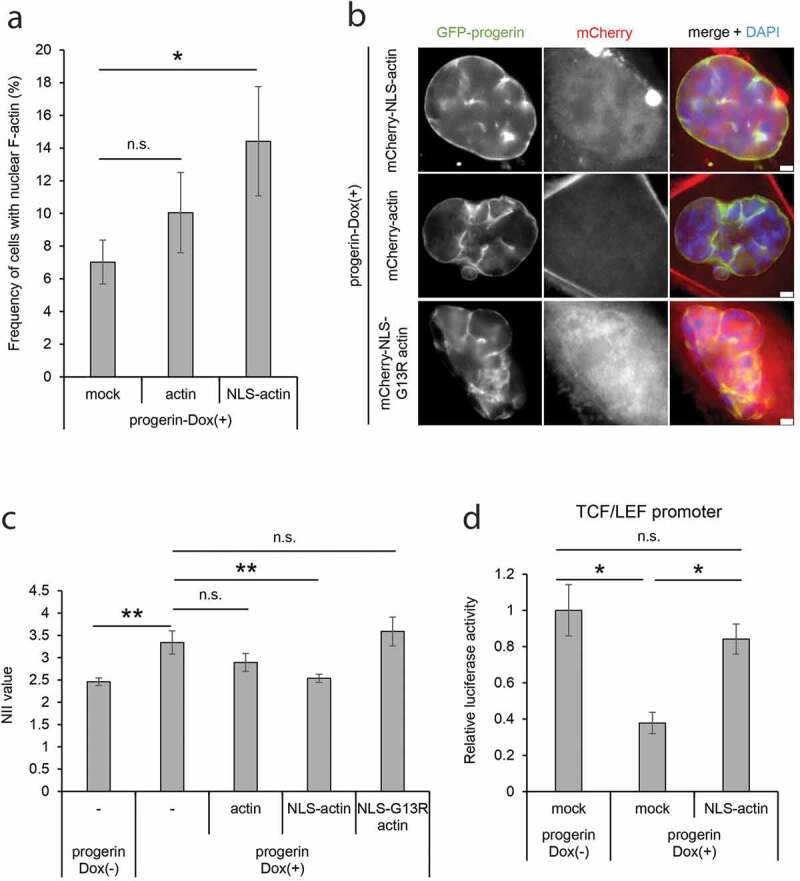
Complementation of HGPS phenotypes by an artificial increase of nuclear F-actin. (a) Frequency of nuclei possessing nuclear F-actin in progerin-expressing cells. The progerin-expressing cells were transfected with a control solution (mock), EYFP-actin (actin), or EYFP-NLS-actin (NLS-actin) together with nAC-mCherry. The mean value of cells transfected with a mock plasmid was defined as 1.0. Data shown are mean ± SEM of four independent experiments. For quantification, 418 cells (mock), 408 cells (actin), and 446 cells (NLS-actin) were analyzed. (b) The nucleus of progerin-expressing cells expressing mCherry-actin, mCherry-NLS-actin, or mCherry-NLS-G13R actin. Images are shown of GFP-progerin, mCherry-actin derivatives, and DAPI-staining. The image of DAPI was merged with GFP and mCherry signals (right panels). Scale bar, 2 μm. (c) The NII value of progerin-Dox(+) cells expressing mCherry-actin (actin), mCherry-NLS-actin (NLS-actin), or mCherry-NLS-G13R actin (NLS-G13R actin). Data shown are mean ± SEM of three independent experiments. For quantification, 526 cells (no transfection, progerin-Dox(-)), 317 cells (no transfection, progerin-Dox(+)), 310 cells (actin), 312 cells (NLS-actin), and 300 cells (NLS-G13R actin) were analyzed. (d) Relative luciferase activity of the TCF/LEF promoter analyzed by luciferase-assay in progerin-Dox(-) and progerin-Dox(+) cells. The cells were transfected with a plasmid expressing mCherry (mock) or mCherry-NLS-actin (NLS-actin). The value in progerin-Dox(-) cells expressing mCherry was assigned as 1.0. Data shown are mean ± SEM of three independent experiments. n.s., not significant; *, *P* < 0.05; **, *P* < 0.01.

HGPS cells show a variety of phenotypes, including nuclear irregularity, transcriptional defects, and DNA-damage repair defects [9,10,12,38,49]. Based on our observation of a reduction of F-actin in the presence of progerin, we hypothesized that the decrease in nuclear F-actin is relevant to some of the HGPS cellular phenotypes. Interestingly, nuclear F-actin is transiently formed at the exit from mitosis, and the appearance of nuclear F-actin is required for chromatin decondensation and proper nuclear expansion [22]. It was also shown that the knockout of β-actin caused impairments in epigenetic landscapes [50,51]. These findings show that the dynamics of polymerization/depolymerization of nuclear actin is required for proper chromatin and nuclear organization. To test the possibility that the decrease of nuclear F-actin leads to irregularity of the nuclear shape in HGPS, we experimentally increased the level of nuclear F-actin by expressing actin fused to a nuclear localization signal (NLS), and analyzed the alteration of nuclear shape of progerin-expressing cells. To confirm the increase of nuclear F-actin formation in this experimental system, nuclear F-actin was observed after the expression of ectopic actin or NLS-actin using the nAC system (Figure 3(a)). The expression of NLS-actin, but not actin lacking an NLS, increased nuclear F-actin by more than 2-fold in comparison to the mock control (Figure 3(a)). Notably, the aberrant nuclear shape of progerin-expressing cells was rescued by the expression of NLS-actin (Figure 3(b), NLS-actin). To quantitatively assess this result, nuclear irregularity was measured and is expressed as the NII value. Whereas the NII value was higher in progerin-expressing cells as compared to the control cells (Suppl. Fig. S1B), the NII value was significantly decreased by the expression of NLS-actin, but not by expression of actin lacking an NLS (Figure 3(c), actin vs. NLS-actin). To test the possibility that NLS-actin indirectly rescued the nuclear shape of progerin-expressing cells through affecting the mitotic defects observed in HGPS [52,53], we analyzed the mitotic phenotypes of the cells after expressing NLS-actin. The consistent appearance of multinucleated cells and cells containing micronuclei even after the expression of NLS-actin excludes this possibility (Suppl. Fig. S5A, S5B). Importantly, the non-polymerizable, nuclear targeted G13R actin mutant failed to rescue the nuclear irregularity of progerin-expressing cells (Figure 3(c), NLS-G13R actin). It has been shown that nuclear actin has roles in regulating general transcriptional activity [50,51,54,55]. To test the possibility that NLS-actin affects nuclear shape through transcriptional alteration of nuclear lamina components, we analyzed the expression of genes for lamin B1, lamin B2, emerin, and lamin A. The expression levels of these genes were not significantly changed by expressing NLS-actin (Suppl. Fig. S6), showing that the rescue of nuclear irregularity in progerin-expressing cells was not caused by the altered levels of these lamina proteins. These observations suggest that the decrease of nuclear F-actin caused by the expression of progerin is, at least in part, responsible for the aberrant nuclear shape of HGPS cells.

The relevance of the reduction in nuclear F-actin to the impairment of Wnt/β-catenin target gene expression in progerin-expressing cells was analyzed using a luciferase assay for the TCF/LEF promoter (Figure 1(c,d)). The expression of NLS-actin, but not actin lacking an NLS, rescued the decline in the activity of the TCF/LEF promoter in progerin-expressing cells (Figure 3(d)). These observations suggest that the reduction of nuclear F-actin is relevant to some cellular phenotypes of HGPS cells.

### Jasplakinolide restores nuclear irregularity in progerin-expressing cells

**Figure 4.**
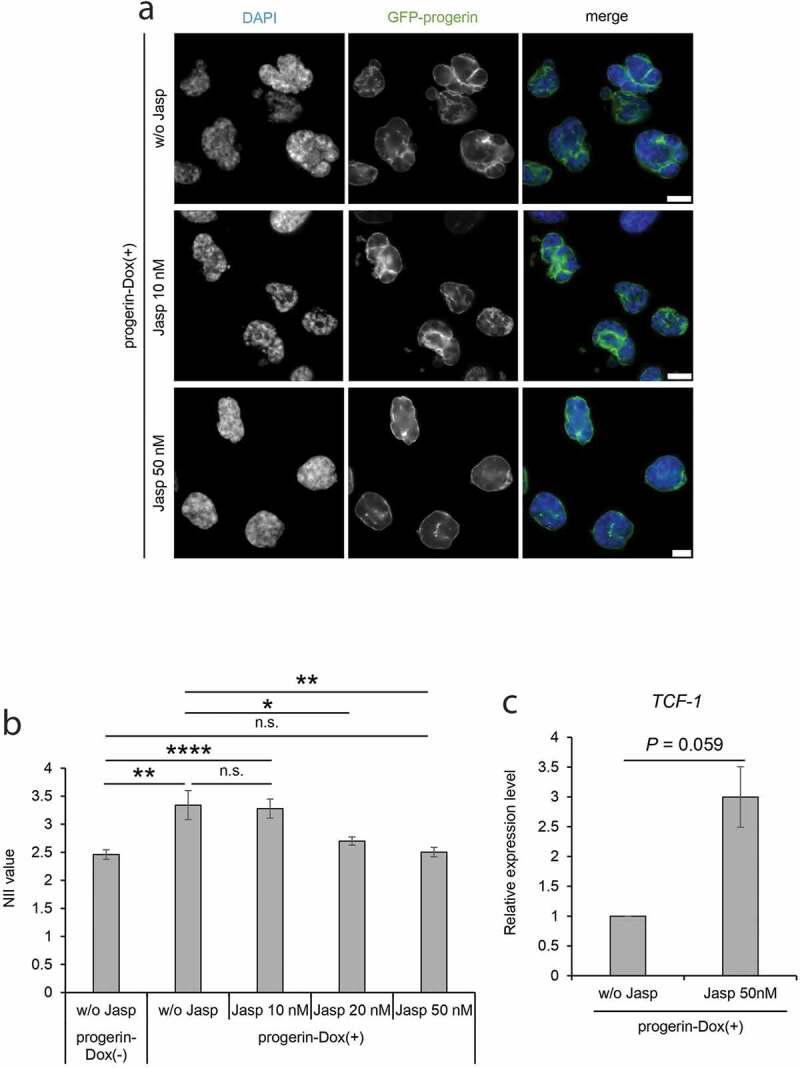
Complementation of nuclear irregularity of progerin-expressing cells by treatment with jasplakinolide. (a) The progerin-expressing cells were treated with 10 nM or 50 nM jasplakinolide for 24 hours, and then stained with DAPI. DAPI and GFP-progerin images of the cells are compared with those of cells not treated with jasplakinolide (w/o Jasp). Scale bar, 10 μm. (b) The NII values of progerin-Dox(-) cells without jasplakinolide treatment (w/o Jasp) and of progerin-Dox(+) cells with or without jasplakinolide treatment (w/o Jasp, 10 nM, 20 nM or 50 nM) were compared. The NII value was determined in each cell group. Error bars indicate SEM of three independent experiments. For quantification, 526 cells (w/o Jasp, progerin-Dox(-)), 317 cells (w/o Jasp, progerin-Dox(+)), 453 cells (Jasp 10 nM), 630 cells (Jasp 20 nM), and 596 cells (Jasp 50 nM) were analyzed in each experiment. (c) The relative expression level of *TCF-1* in progerin-expressing cells treated with jasplakinolide. The expression level was measured by qRT-PCR and was normalized with respect to that of *GAPDH* gene. The expression level of *TCF-1* in progerin-Dox(+) without jasplakinolide treatment was assigned as 1.0. Data shown are mean ± SEM of three independent experiments. n.s., not significant; *, *P*<0.05; **, *P* < 0.01; ****, *P* < 0.0001.

Jasplakinolide is a compound that induces actin polymerization [56,57], and is reported to be a candidate therapeutic drug for the treatment of human cancer [58,59]. We hypothesized that jasplakinolide could facilitate nuclear F-actin formation and rescue cellular phenotypes in HGPS. When we treated cultured NIH3T3 cells expressing nAC with jasplakinolide for 12 hours, an increase in nuclear F-actin was observed (Suppl. Fig. S7A, S7B), indicating that jasplakinolide facilitates F-actin formation not only in the cytoplasm but also in the nucleus. Interestingly, we observed that jasplakinolide treatment for 12 hours rescued the distorted nuclear shape of progerin-Dox(+) cells in a dose-dependent manner (Figure 4(a)). The quantitative analysis of the nuclear irregularity using the NII value revealed a statistically significant rescue of the nuclear shape upon jasplakinolide treatment (Figure 4(b)). We also analyzed the expression level of the *TCF-1* gene in progerin-Dox(+) cells with or without the jasplakinolide treatment. Whereas the expression of *TCF-1* is reduced in progerin-Dox(+) cells (Figure 1(d)), it was observed that the jasplakinolide treatment increased the expression of *TCF-1* (Figure 4(c)). These results suggest that jasplakinolide may be used to diminish the severity of HGPS phenotypes through modulation of nuclear F-actin.

### Nuclear F-actin and the molecular etiology of HGPS cells

In this study, we have shown that nuclear F-actin is reduced in progerin-expressing cells and that the experimental increase in nuclear F-actin level diminishes the severity of some of the cellular phenotypes in HGPS cells. The phenotypes that are diminished by the increase of nuclear F-actin can be explained by the roles of nuclear F-actin in the organization of nuclear shape and in the expression of Wnt/β-catenin-targeting genes.

**Figure 5.**
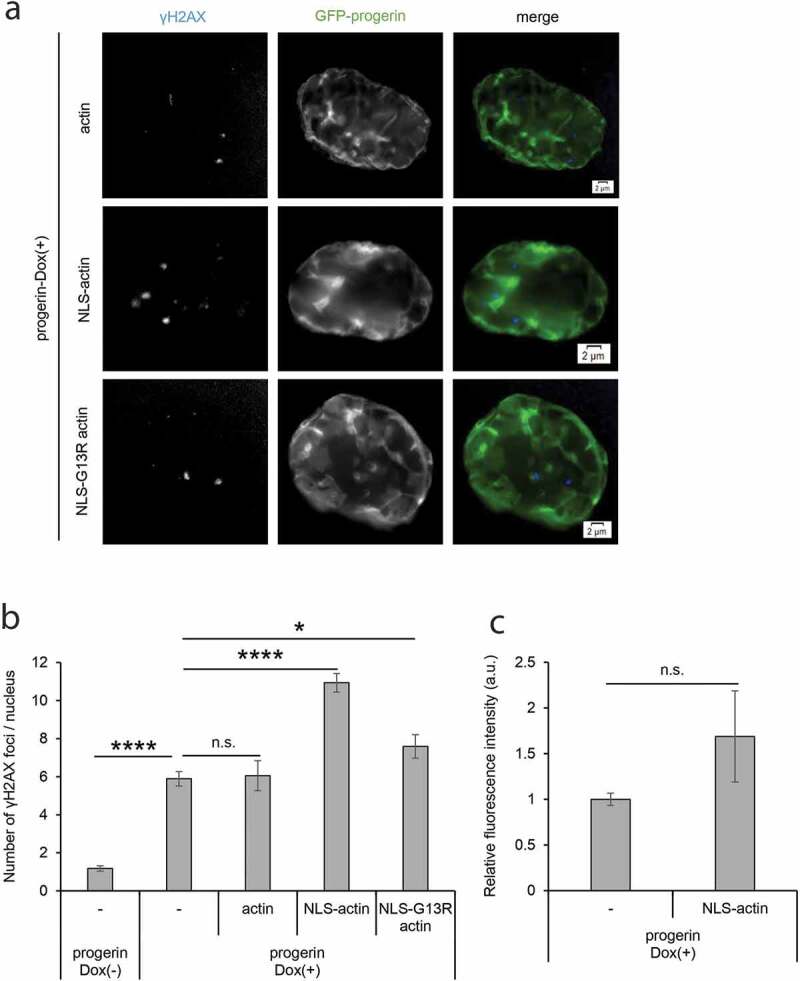
Effects of inducing artificial nuclear F-actin in DNA damage repair and cell senescence. (a) γH2AX foci in progerin-expressing cells expressing mCherry-actin, mCherry-NLS-actin, or mCherry-NLS-G13R actin were detected by fluorescence microscopy. Representative images of γH2AX and GFP-progerin are shown. Scale bar, 2 μm. (b) The number of γH2AX foci per nucleus in progerin-Dox(-) and progerin-Dox(+) cells. The latter cells expressing mCherry-actin (actin), mCherry-NLS-actin (NLS-actin), or mCherry-NLS-G13R actin (NLS-G13R actin) were also analyzed. Data shown are mean ± SEM of three independent experiments, and 100 cells were analyzed in each experiment. (c) The relative signal intensity of SA-β-gal (arbitrary units) in progerin-expressing cells with or without the expression of mCherry-NLS-actin. The relative intensity of progerin-expressing cells without mCherry-NLS-actin was assigned as 1.0. Data shown are mean ± SEM of three independent experiments. n.s., not significant; *, *P* < 0.05; ****, *P* < 0.0001.

In addition, nuclear F-actin is required for DNA double-strand damage (DSB) repair [24,25], and DSB repair is generally impaired in HGPS patients cells [9,12]. These observations imply the possibility that, as well as the irregularity of the nuclear shape, the defect in DNA damage repair may be rescued by the artificial increase of nuclear F-actin. However, the expression of NLS-actin in progerin-expressing cells increased rather than decreased, the number of γH2AX foci in the nuclei (Figure 5(a,b)). Interestingly, the expression of NLS-G13R actin, which has a negligible ability to form F-actin, did not affect the number of γH2AX foci. These results suggest that, whereas nuclear F-actin affects the DSB repair in HGPS, the reduction in the total amount of nuclear F-actin itself cannot explain the defect of DSB repair. It is plausible that the dynamic regulation and turnover of F-actin formation at particular locations within the nucleus, but not the total amount of nuclear F-actin, is required to achieve proper DSB repair. As well as the DSB repair activity, the SA-β-gal activity which is a marker of cellular senescence of HGPS cells was not rescued by expressing NLS-actin (Figure 5(c)), supporting the notion that not all aspects of the HGPS phenotype are a function of a reduction in the total amount of nuclear F-actin.

Taken together, our study reveals a novel relationship between nuclear actin and HGPS. Recent research has shown that nuclear actin is involved in multiple diseases, including cancer and myopathy [60,61]. Understanding the molecular mechanisms of nuclear actin in diseases by using model systems could contribute to the possibility of using known actin-binding chemicals for therapeutic purposes.

## Supplementary Material

Supplemental Material
