# Supplementary material for: Impairment of nuclear F-actin formation and its relevance to cellular phenotypes in Hutchinson-Gilford progeria syndrome
Source: Nucleus. 2020 Sep 20;11(1):250–63. doi: 10.1080/19491034.2020.1815395 (PMC7529414; doi:10.1080/19491034.2020.1815395)
Supplement: Supplemental Material [file KNCL_A_1815395_SM4086.pdf]

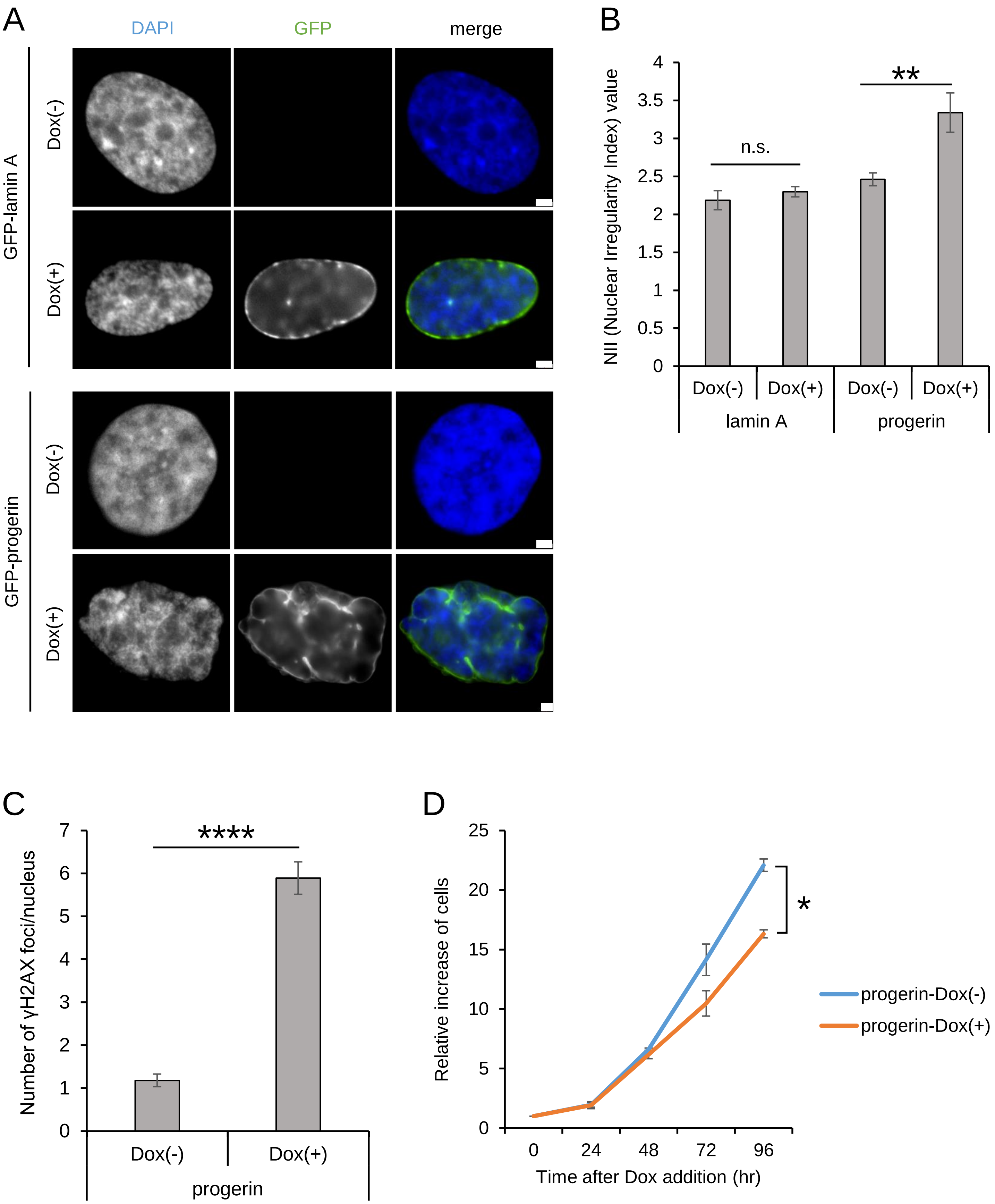
A
DAPI
GFP
merge
GFP-lamin A
Dox(-)
Dox(+)
GFP-progerin
Dox(-)
Dox(+)
B
NII (Nuclear Irregularity Index) value
n.s.
**
Dox(-)
Dox(+)
Dox(-)
Dox(+)
lamin A
progerin
C
****
Number of γH2AX foci/nucleus
Dox(-)
Dox(+)
progerin
D
Relative increase of cells
*
progerin-Dox(-)
progerin-Dox(+)
Time after Dox addition (hr)

Suppl. Fig. S1

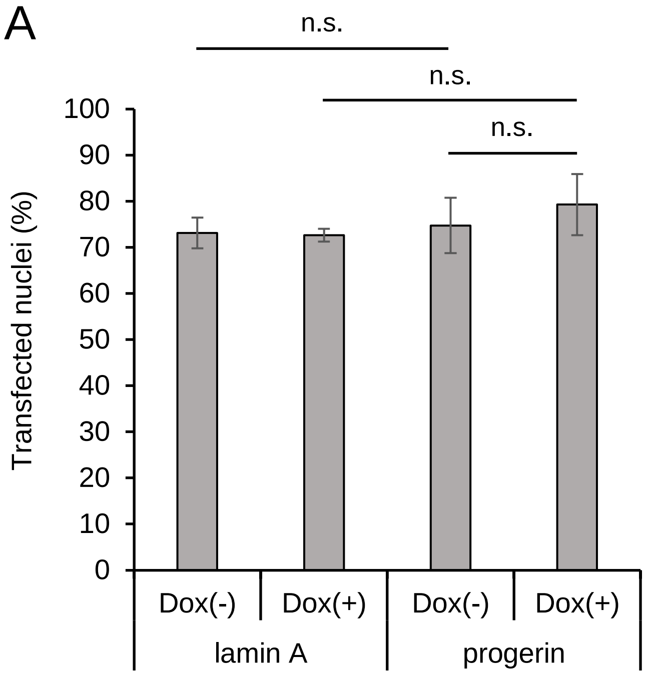
A
n.s.
n.s.
n.s.
Transfected nuclei (%)
100
90
80
70
60
50
40
30
20
10
0
Dox(-)
Dox(+)
Dox(-)
Dox(+)
lamin A
progerin

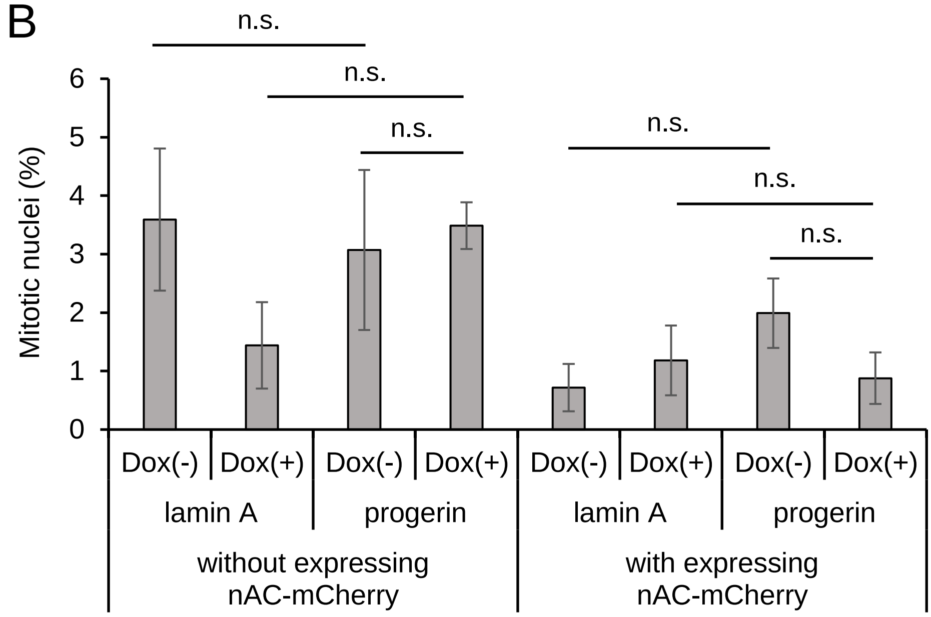
B
n.s.
n.s.
n.s.
n.s.
n.s.
n.s.
Mitotic nuclei (%)
6
5
4
3
2
1
0
Dox(-)
Dox(+)
Dox(-)
Dox(+)
Dox(-)
Dox(+)
Dox(-)
Dox(+)
lamin A
progerin
lamin A
progerin
without expressing nAC-mCherry
with expressing nAC-mCherry

Suppl. Fig. S2

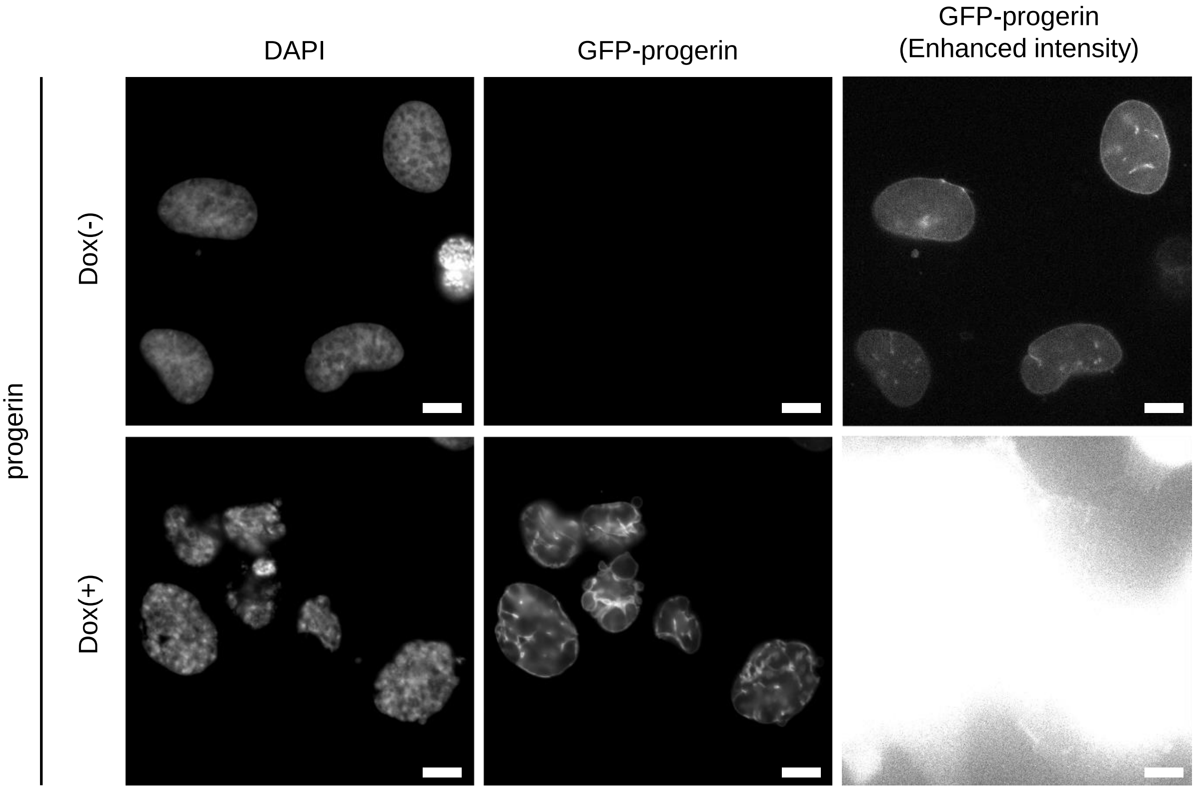
DAPI
GFP-progerin
GFP-progerin
(Enhanced intensity)
progerin
Dox(-)
Dox(+)

Suppl. Fig. S3

A

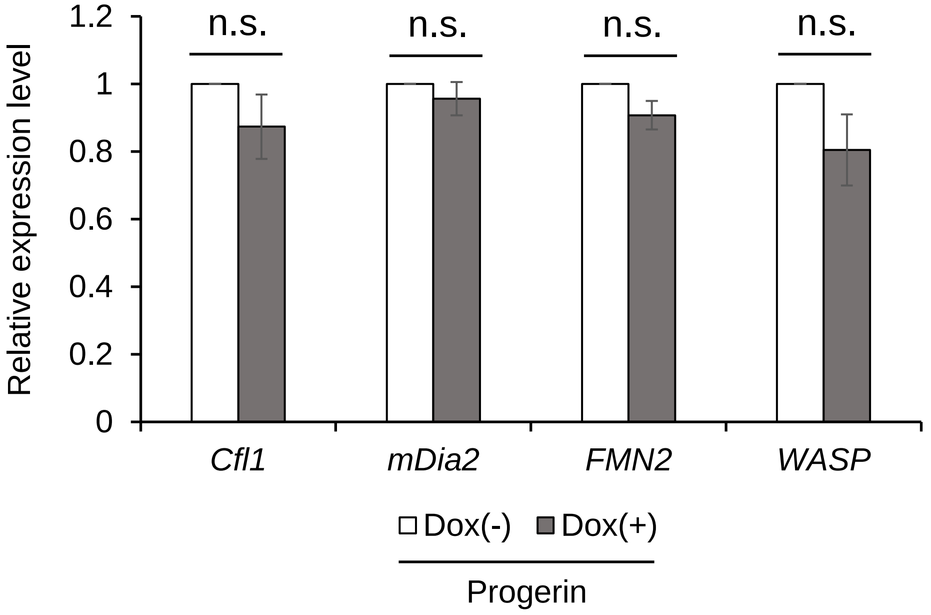

B

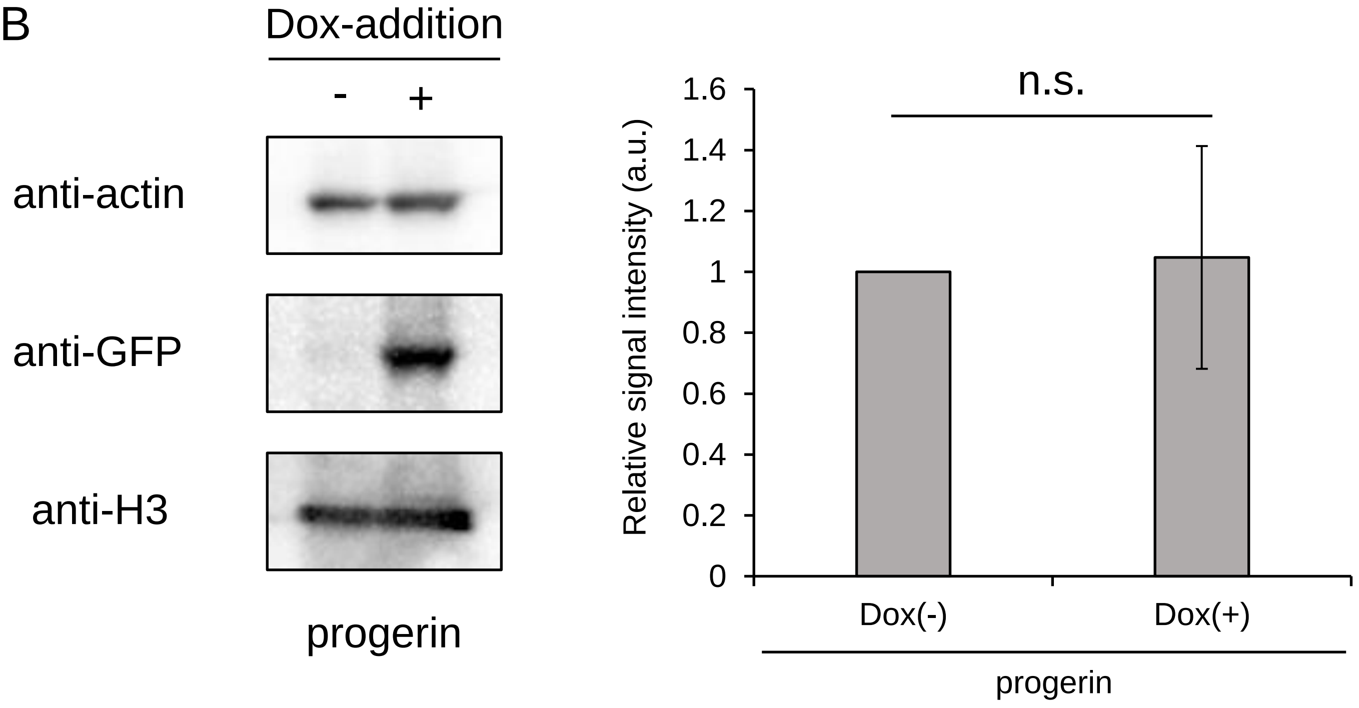

Suppl. Fig. S4

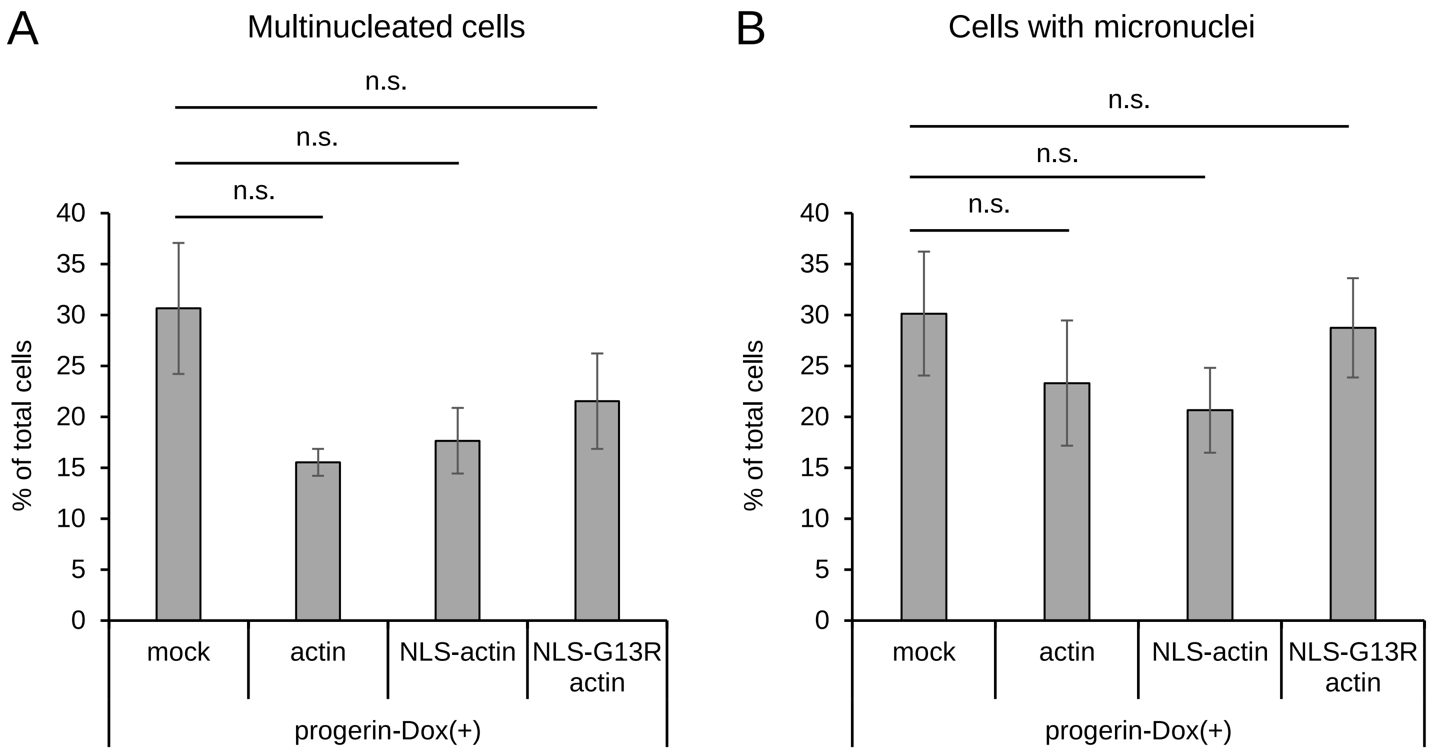
A
Multinucleated cells
n.s.
n.s.
n.s.
% of total cells
40
35
30
25
20
15
10
5
0
mock
actin
NLS-actin
NLS-G13R actin
progerin-Dox(+)
B
Cells with micronuclei
n.s.
n.s.
n.s.
% of total cells
40
35
30
25
20
15
10
5
0
mock
actin
NLS-actin
NLS-G13R actin
progerin-Dox(+)

Suppl. Fig. S5

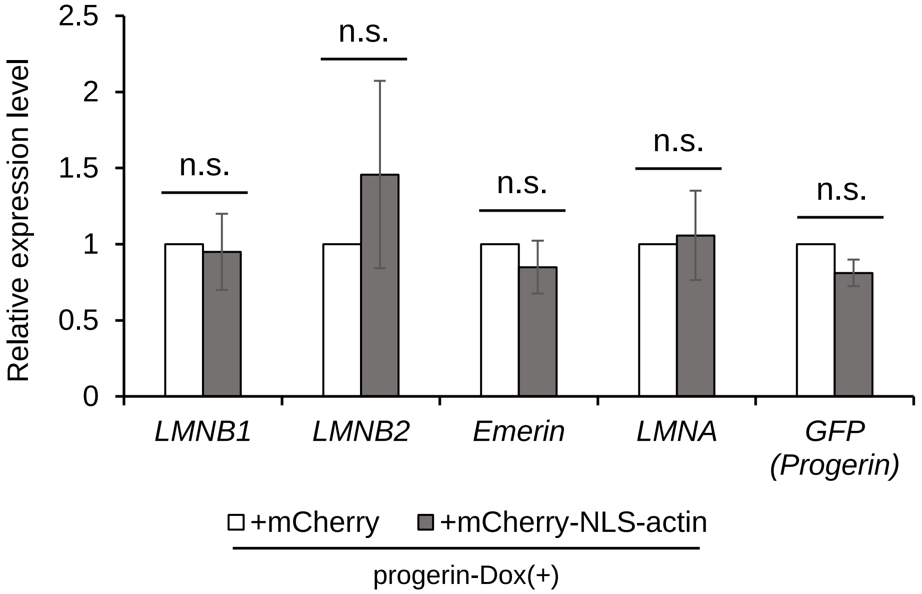
Relative expression level
2.5
2
1.5
1
0.5
0
n.s.
n.s.
n.s.
n.s.
n.s.
LMNB1
LMNB2
Emerin
LMNA
GFP (Progerin)
+mCherry
+mCherry-NLS-actin
progerin-Dox(+)

Suppl. Fig. S6

A

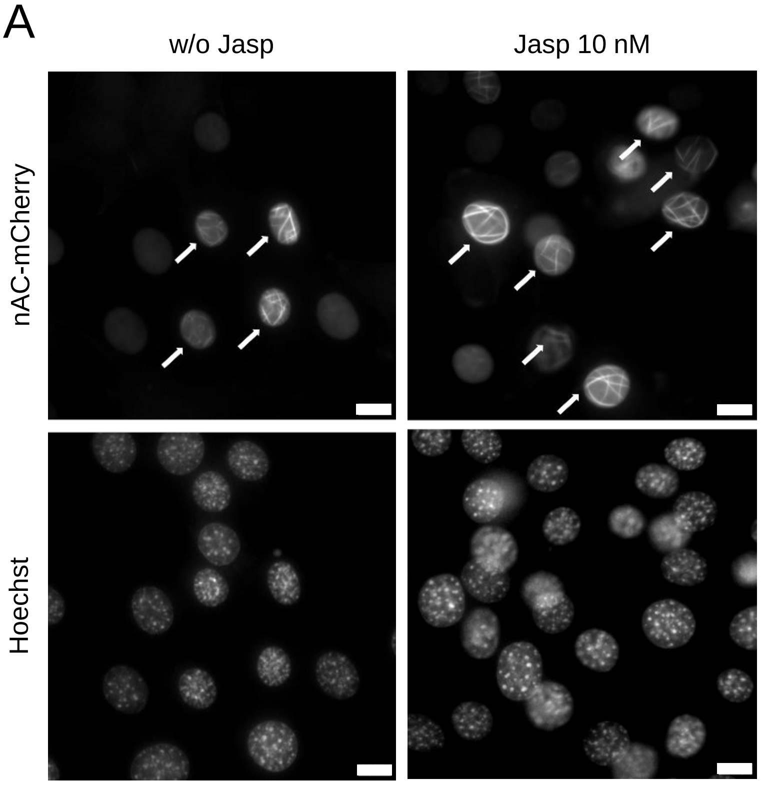

B

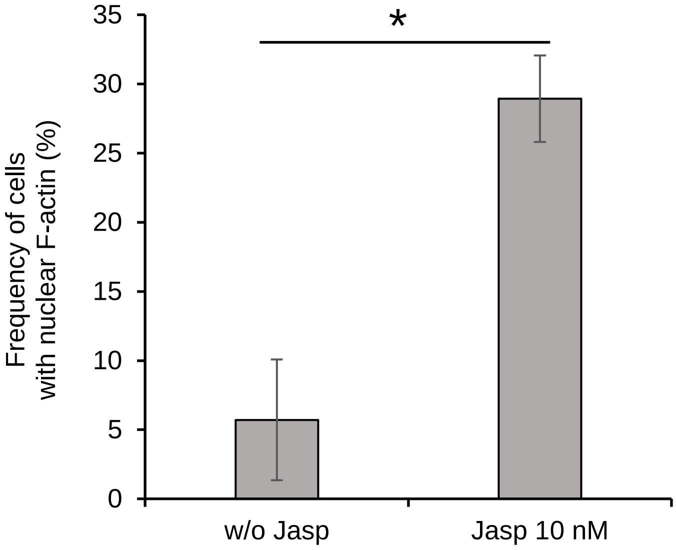

Suppl. Fig. S7

SUPPLEMENTARY FIGURE LEGENDS

Supplementary Fig. S1. (A) Human dermal fibroblast cells (HDF) containing GFP-lamin A or GFP-progerin under control of a Dox-inducible promoter were cultured with or without Dox and DAPI stained. Scale bar, 2 µm. (B) The NII values of the DAPI-stained nuclei observed as in A were compared. Data shown are mean ± SEM of three independent experiments. For quantification, 316 cells (lamin A-Dox(-)), 392 cells (lamin A-Dox(+)), 526 cells (progerin-Dox(-)), and 317 cells (progerin-Dox(+)) were analyzed in each experiment. (C) The number of γH2AX foci per nucleus of cells with or without progerin expression is compared. Data shown are mean ± SEM of three independent experiments, and 100 cells were analyzed in each experiment. (D) The relative growth of progerin-expressing cells. The number of living cells was measured using the trypan blue exclusion method. The mean number of progerin-Dox(-) cells was defined as 1.0. Data shown are mean ± SEM of three independent experiments. n.s., not significant; *, $P < 0.05$; **, $P < 0.01$; ****, $P < 0.0001$.

Supplementary Fig. S2. (A) Transfection efficiency of cells transfected with the plasmid for expression of nAC-mCherry. Transfected cells were identified by the detection of mCherry fluorescence under a microscope. Data shown are mean ± SEM of three independent experiments. For quantification, 543 cells (lamin A-Dox(-)), 462 cells (lamin A-Dox(+)), 559 cells (progerin-Dox(-)), and 600 cells (progerin-Dox(+)) were analyzed. (B) Percentage of mitotic cells with or without expressing of nAC-mCherry. Mitotic cells were identified by immunofluorescence with an anti-α-tubulin antibody. Data shown are mean ± SEM of three independent experiments. For quantification, 346 cells (lamin A-Dox(-)), 350 cells (lamin A-Dox(+)), 329 cells (progerin-Dox(-)), and 344 cells (progerin-Dox(+)) were analyzed in cells without nAC-mCherry expression. In cells with nAC-mCherry expression, 397 cells (lamin A-Dox(-)), 336 cells (lamin A-Dox(+)), 413 cells (progerin-Dox(-)), and 479 cells (progerin-Dox(+)) were analyzed. n.s., not significant.

Supplementary Fig. S3. Expression of GFP-progerin under uninduced (Dox(-)) and induced (Dox(+)) conditions. After DAPI staining of the cells, the fluorescence of DAPI and GFP was observed under a microscope. In the panel of "enhanced intensity", low-intensity pixels were enhanced using an analytical device. Scale bar, 10 μm.

Supplementary Fig. S4. (A) The relative expression level of cofilin1 (*Cfl1*), mDia2, formin2 *(FMN2)*, and WASP was measured by qRT-PCR and was normalized with respect to that of *GAPDH* gene. The expression level of each gene in progerin-Dox(-) was assigned as 1.0. Data shown are mean ± SEM of three independent experiments. n.s., not significant. (B) The total amount of actin in whole-cell extracts of progerin-expressing cells was analyzed by western blotting with anti-actin, anti-GFP, and anti-histone H3 (H3) antibodies. A representative image of western blotting analysis is shown in the left panel. The relative signal intensity of actin normalized with the H3 signal intensity is shown in the right panel. The mean value of progerin-Dox(-) cells was defined as 1.0, and data shown are mean ± SEM of three independent experiments.

Supplementary Fig. S5. The appearance of multinucleated cells (A) and of cells possessing micronuclei (B) was observed in progerin-expressing cells. Cells were transfected with a plasmid expressing mCherry (mock), mCherry-actin (actin), mCherry-NLS-actin (NLS-actin), or mCherry-NLS-G13R actin (NLS-G13R actin). Cells possessing multiple nuclei were defined as multinucleated cells. Micronuclei were defined as the presence of small additive nuclei. Data shown are mean ± SEM of three independent experiments. For quantification, 333 cells (mock), 309 cells (actin), 359 cells (NLS-actin), and 307 cells (NLS-G13R-actin) were analyzed.

Supplementary Fig. S6. The relative expression levels of lamin B1 (*LMNB1*), lamin B2 (*LMNB2*), emerin*,* lamin A (*LMNA*), and GFP were analyzed by qRT-PCR. The qPCR values of these genes were normalized with respect to that of the *GAPDH* gene. The expression level of each gene in progerin-Dox(+) expressed mCherry was assigned as 1.0. Data shown are mean ± SEM of three independent experiments. n.s., not significant.

Supplementary Fig. S7. (A) Nuclear F-actin was observed in NIH3T3 cells stably expressing nAC-mCherry with or without 10 nM jasplakinolide (Jasp) for 12 hours. The nucleus of the cells was visualized with Hoechst 33342. Arrows indicate nuclei possessing nuclear F-actin. Scale bar, 10 μm. (B) Percentages of cells possessing nuclear F-actin are compared between the cells with or without the 10 nM jasplakinolide treatment. Data shown are mean ± SEM of three independent experiments. For quantification, 907 cells (w/o Jasp), and 1007 cells (Jasp 10 nM) were analyzed in each experiment. *, $P < 0.05$.
